# Evolution of surface-based deformable image registration for adaptive radiotherapy of non-small cell lung cancer (NSCLC)

**DOI:** 10.1186/1748-717X-4-68

**Published:** 2009-12-21

**Authors:** Matthias Guckenberger, Kurt Baier, Anne Richter, Juergen Wilbert, Michael Flentje

**Affiliations:** 1 Department of Radiation Oncology, University of Wuerzburg, Wuerzburg, Germany

## Abstract

**Background:**

To evaluate the performance of surface-based deformable image registration (DR) for adaptive radiotherapy of non-small cell lung cancer (NSCLC).

**Methods:**

Based on 13 patients with locally advanced NSCLC, CT images acquired at treatment planning, midway and the end of the radio- (n = 1) or radiochemotherapy (n = 12) course were used for evaluation of DR. All CT images were manually [gross tumor volume (GTV)] and automatically [organs-at-risk (OAR) lung, spinal cord, vertebral spine, trachea, aorta, outline] segmented. Contours were transformed into 3D meshes using the Pinnacle treatment planning system and corresponding mesh points defined control points for DR with interpolation within the structures. Using these deformation maps, follow-up CT images were transformed into the planning images and compared with the original planning CT images.

**Results:**

A progressive tumor shrinkage was observed with median GTV volumes of 170 cm^3 ^(range 42 cm^3 ^- 353 cm^3^), 124 cm^3 ^(19 cm^3 ^- 325 cm^3^) and 100 cm^3 ^(10 cm^3 ^- 270 cm^3^) at treatment planning, mid-way and at the end of treatment. Without DR, correlation coefficients (CC) were 0.76 ± 0.11 and 0.74 ± 0.10 for comparison of the planning CT and the CT images acquired mid-way and at the end of treatment, respectively; DR significantly improved the CC to 0.88 ± 0.03 and 0.86 ± 0.05 (p = 0.001), respectively. With manual landmark registration as reference, DR reduced uncertainties on the GTV surface from 11.8 mm ± 5.1 mm to 2.9 mm ± 1.2 mm. Regarding the carina and intrapulmonary vessel bifurcations, DR reduced uncertainties by about 40% with residual errors of 4 mm to 6 mm on average. Severe deformation artefacts were observed in patients with resolving atelectasis and pleural effusion, in one patient, where the tumor was located around large bronchi and separate segmentation of the GTV and OARs was not possible, and in one patient, where no clear shrinkage but more a decay of the tumor was observed.

**Discussion:**

The surface-based DR performed accurately for the majority of the patients with locally advanced NSCLC. However, morphological response patterns were identified, where results of the surface-based DR are uncertain.

## Background

Traditionally, radiotherapy was characterized by a unidirectional work-flow: planning images were acquired prior to treatment, these images were the basis for generation of radiotherapy treatment plans and these plans were delivered throughout the total course of radiotherapy. For certain indications, a shrinking field approach was practiced but delineation of the boost target volume was still performed in the primary planning image.

Recently, volume imaging became available for in-room image guidance aiming at verification of the target position prior to treatment. Techniques like in-room CT scanner [1], cone-beam CT (both kilovoltage [2] and megavoltage [3] cone beam CT) and the tomotherapy system [4] offer sufficient soft tissue contrast for position verification of soft tissue tumors. Studies using these imaging technologies clearly showed that the planning CT image needs to be considered as a snapshot of the patients' anatomy, which may or may not be representative for the course of fractionated radiotherapy. For pulmonary tumors, base-line drifts independently from the bony anatomy have been reported [5-7], which may decrease target coverage and increase doses to organs-at-risk (OAR) if not corrected by means of image guidance.

Analysis of these verification images acquired during radiotherapy showed not only changes of the target position but also more complex changes like weight loss of the patients during treatment, changes of pulmonary atelectasis and pleural effusion and tumor shrinkage. Barker et al. reported regression of irradiated head and neck tumors by 70% during the treatment course and this tumor shrinkage was associated with changes of the spatial relationship between the target and the parotid glands [8]. Similar findings were made for non-small-cell lung cancer (NSCLC), where a continuous tumor regression during radiotherapy was observed [9].

This continuous tumor regression during radiotherapy makes adaptive radiotherapy (ART) approaches highly attractive: adaptive radiation therapy is defined as a closed-loop, iterative process where the treatment plan is modified based on feedback measurements performed during treatment [10]. Such concepts aim at improved accuracy of treatment allowing either an escalation of the irradiation dose or reduction of doses to OAR e.g. by shrinking the radiation fields corresponding to target shrinkage. Additionally, adaptation of the treatment plan to tumor progression or systematic target displacements during treatment are expected to improve target coverage. If multiple plans are delivered during the course of treatment, calculation of composite dose distributions is required for inclusion of this information into the feedback loop of ART and for final analysis of the delivered dose distribution. In the absence of morphological changes, time weighted summation of these dose distributions is quite straight forward. However, if ART is based on images with significant morphological changes of the patients' anatomy, deformable image registration is required for tracking of each anatomical structure, of all corresponding voxels. The vectors between corresponding voxels define deformation maps, which are finally applied to the corresponding dose distributions and allow for their summation. Consequently, deformable image registration (DR) is an essential part of all ART protocols, where morphological changes may be present. Additionally, even if one single treatment plan is delivered during the total course of radiotherapy, the uncertainties described above make the data of the initial treatment plan with doses to the target and OARs unreliable.

This study evaluates a DR algorithm to account for shrinkage of NSCLC during primary radiochemotherapy. CT images were acquired mid-way and at the end of the radiotherapy course and these CT images were registered with the planning CT image. The DR algorithm requires (automatic and manual) segmentation of all images and the deformation map is based on corresponding surface points. The accuracy of this DR approach was analyzed and limitations were evaluated.

## Materials and methods

This study is based on 13 patients treated with radiotherapy (n = 1) or simultaneous radiochemotherapy (n = 12) for primary, advanced stage NSCLC. Seven patients were enrolled in a randomized phase III trial, where conventionally fractionated radiotherapy was combined with chemotherapy of cisplatin and oral vinorelbine; five additional patients were treated with the same radiotherapy and chemotherapy protocol. Simultaneous chemotherapy was refused by one patient, who was treated with radiotherapy only. Written informed consent was obtained by all patients. Details of patient and treatment characteristics are listed in table 1.

**Table 1:** Patient characteristics: squamous cell carcinoma (SSC), superior-inferior direction (SI), anterior-posterior direction (AP), cisplatin (DDP)

Patient	Age (years)	Clinical T N stage	Stage	Histology	Motion amplitude in SI direction (mm)	GTV volume in planning CT (cm^3^)	Single dose (Gy)	Total dose (Gy)	Simultaneous chemotherapy
Pat #1	48,8	T4 N2	IIIB	Adeno Ca	< 5	42.2	2	66	DDP + Navelbine
Pat #2	52,6	T3 N3	IIIB	Adeno Ca	12	60	2	66	DDP + Navelbine
Pat #3	71,5	T4 N2	IIIB	SSC	< 5 (5 mm AP)	62.1	2	70	DDP + Navelbine
Pat #4	56,8	T3 N1	IIIA	SSC	14	109.1	2	62	DDP + Navelbine
Pat #5	49,0	T2 N2	IIIA	Adeno Ca	11	147.2	2	66	DDP + Navelbine
Pat #6	34,9	T4 N2	IIIB	Adeno Ca	< 5	160.1	2	70	DDP + Navelbine
Pat #7	74,2	T4 N2	IIIB	Adeno Ca	< 5	169.3	2	66	DDP + Navelbine
Pat #8	75,2	T4 N1	IIIA	Adeno Ca	< 5	202.9	2	66	DDP + Navelbine
Pat #9	61,7	T3 N2	IIIA	Undifferentiated NSCLC	10	2	216.1	66	DDP + Navelbine
Pat #10	82,1	T4 N2	IIIB	Adeno Ca	< 5	225.2	2	66	none
Pat #11	57,6	T4 N3	IIIB	Neuroendocrine Ca	< 5	302.8	2	66	DDP + Navelbine
Pat #12	63,5	T4 N3	IIIB	Undifferentiated NSCLC	< 5	352.0	2	66	DDP + Navelbine
Pat #13	68,8	T2 N2	IIIA	SSC	< 5	382.5	2	70	DDP + Navelbine

For treatment planning, a conventional 3D CT study with 5 mm slice thickness was acquired for all patients using a 24-slice CT scanner (Somatom Sensation Open; Siemens Medical Solutions, Erlangen, Germany). Midway through treatment [median 21^st ^day after start of treatment (19 - 24)] and in the sixth week of treatment [median 43^rd ^day after start of treatment (40 - 47)], a follow-up CT scan was performed; patients were positioned in the same way as at treatment planning and treatment delivery.

All CT images were imported into the Pinnacle treatment planning system, research version 8.9 (Philips Radiation Oncology Systems, Fitchburg, WI, USA). Images were registered using rigid automatic image registration in six degrees of freedom with the region of interest for image registration confined to the thoracic vertebral spine. Lungs, spinal cord and the patients' outline were delineated using automatic image segmentation. If the target volumes were close to vertebral column (n = 11), the trachea (n = 11), the aortic artery (n = 4) or the sternum (n = 1), these structures were additionally delineated using semiautomatic segmentation: the structures were manually delineated in the planning CT series, then propagated into the follow-up CT images and their shape and position were adjusted automatically within the Pinnacle software [11].

The macroscopic primary tumor was delineated as the gross tumor volume (GTV_primary_) in the CT pulmonary window of the planning CT image; the soft tissue window was used for delineation if the tumor was located adjacent to the thoracic wall and to the mediastinum. Pathologically enlarged lymph nodes were included into this GTV_primary _if separation of the primary tumor and lymph node metastases was not possible (n = 11). Lymph node metastases were located distant to the primary tumors in two patients and these lymph node metastases were delineated as GTV_LN_. These GTV structures were propagated into the follow-up CT images and the structures were adjusted manually to account for changes of tumor position, shape and size.

### Deformable Image Registration

**Figure 1:**
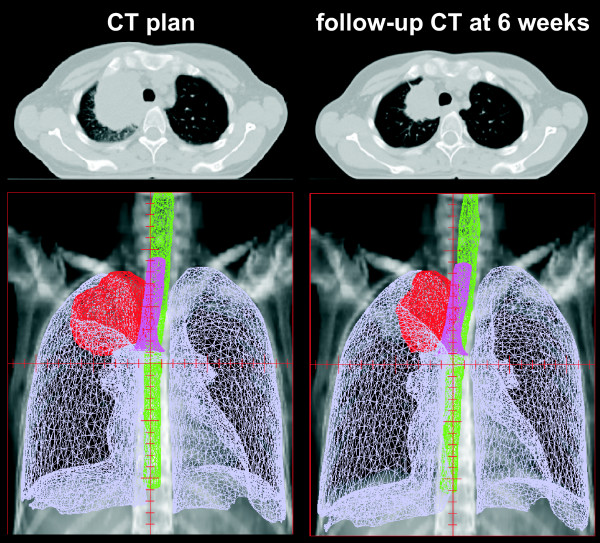
**Planning CT image and follow-up CT image acquired at week 6 of combined radiochemotherapy for patient #6: corresponding three-dimensional meshes of the GTV, lungs, spinal cord and trachea are displayed in the second row**.

Prior to propagation and adaptation of the planning structures in the follow-up CT images, all structures were converted into 3D meshes: a mesh consists of vertices located on the organ surface, connected by edges to neighbouring triangles. These meshes were the basis for DR of the primary planning CT image and all follow-up CT images. In Pinnacle TPS a surface/model based DR is implemented [12-15]: the deformation of a particular location on the surface of one region of interest (ROI) is measured from a vertex of the mesh in the reference data set to the corresponding vertex in the secondary data set. The set of all corresponding mesh vertices from all structures (control points of the deformation algorithm) defines a surface deformation (Fig. 1). A deformation model [elastic body splines (EBS), Gauss algorithm, Poisson's ratio of the elastic deformation (Nu) set to 0.3] then interpolates the surface deformation to the entire volume to derive a volumetric deformation field. The deformation map was then applied to the follow-up CT image; in case of a perfect DR, the deformed follow-up image should then be identical to the planning CT image.

Follow-up CT images acquired mid-way through the radiotherapy series and at the end of radiotherapy were deformed to the corresponding planning CT images. Mesh points from GTV structures and all normal tissue structures were selected for DR.

### Evaluation of Deformable Image Registration

Visual evaluation of planning CT images (CT_plan_), follow-up CT images (CT_FU_) and follow-up CT images deformed to the planning CT image (CT_deform_) was performed. CT_plan _and CT_FU _were compared regarding the location of normal tissue landmark structures in the lung (small vessels and bronchi) in relationship to the shrinking tumor. A fixed position of these landmark structures in CT_plan _and CT_FU _despite tumor shrinkage during radiotherapy would suggest that the tumor had grown in an infiltrative pattern within the pulmonary structure. A change of the position of these landmark structures towards the shrinking tumor in the CT_FU _would suggest an expansive, displacing growth pattern.

For quantitative analysis of the DR, all images were imported into in-house software. Two CT image series were loaded into this software and manual registration of these image data sets was performed with the registration based on the bony spine. A cubic region of interest (ROI) was defined for analysis of the differences between the two image series. Two different ROIs were analyzed. ROI_extended _covered the GTV in superior-inferior direction plus 10 mm but included the whole body contour in axial directions. ROI_limited _covered the GTV plus 10 mm in all directions. The Pearson's correlation coefficient (CC) was calculated for corresponding voxels based on ROI_extended _and ROI_limited _and this was used as a parameter for the similarity between the two image data sets.

Additionally, a landmark-based evaluation of the DR was performed in the Pinnacle planning system. Corresponding landmark structures were identified manually between CT_plan _and CT_FU _and between CT_plan _and CT_deform _and the 3D distances between corresponding landmark points were calculated; this analysis was limited to the CT_FU _acquired in the sixth week of treatment. Four different sets of anatomical landmarks were analyzed:

1. Most anterior, posterior, left, right, superior and inferior position of the GTV

2. Carina

3. Bifurcations of intra-pulmonary vessels in the same lobe as the NSCLC; analysis of four to five landmark structures was intended

4. Bifurcations of intra-pulmonary vessels in the different lobes compared to the NSCLC but in the same lung; analysis of four to five landmark structures was intended

### Statistical analysis

Statistica 7.0 was utilized for statistical analysis (Statsoft, Tulsa, OK, USA). Mann-Whitney-U test was performed for comparison of two subset analyses and Wilcoxon test was used for matched pair analyses. The differences were considered significant for p < 0.05.

## Results

### Quantification of tumor regression

Median volume of the GTV in the planning CT images was 170 cm^3 ^(range 25 cm^3 ^- 353 cm^3^); the GTV volume decreased to median 124 cm^3 ^(19 cm^3 ^- 325 cm^3^) and 100 cm^3 ^(10 cm^3 ^- 270 cm^3^) mid-way and at the end of radio-chemotherapy.

**Figure 2:**
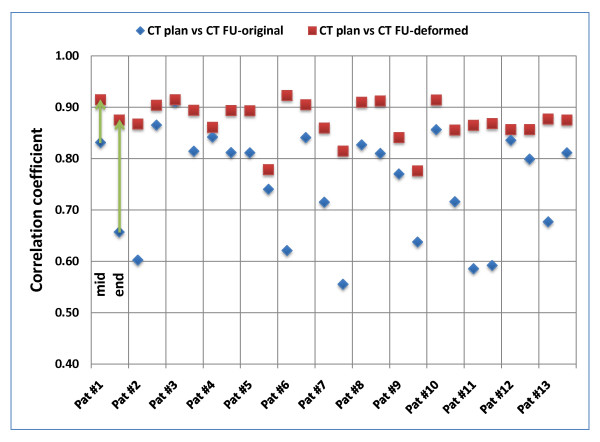
**Patient individual voxel-based analysis of deformable image registration based on ROI_extended _(covering the GTV + 10 mm in superior-inferior direction but including the whole body contour in axial directions)**. Correlation-coefficients (CC) were calculated between the planning CT image and original/deformed follow-up CT image (mid-way and at the end of the treatment course).

**Figure 3:**
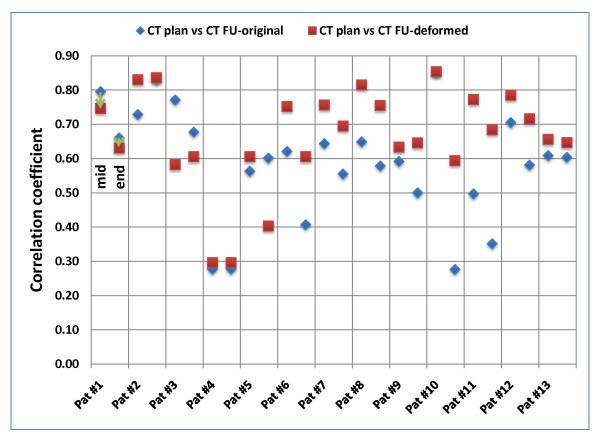
**Patient individual voxel-based analysis of deformable image registration based on ROI_limited _(covering the GTV plus 10 mm in all directions)**. Correlation-coefficients (CC) were calculated between the planning CT image and original/deformed follow-up CT image (mid-way and at the end of the treatment course).

Comparison of CT_plan _and CT_FU _was performed for quantification of anatomical changes during the treatment course. Based on ROI_extended_, CC was 0.76 ± 0.11 and 0.74 ± 0.10 for comparison of CT_plan _and the CT_FU _acquired mid-way and at the end of treatment, respectively (Fig. 2). If the analysis was based on ROI_limited_, CC was decreased with 0.64 ± 0.15 and 0.53 ± 0.16 mid-way and at the end of treatment, respectively (Fig. 3). These values indicate progressive changes of the patients' anatomy and GTV volume and shape during treatment.

For ROI_limited_, absolute reduction of the GTV volume between CT_plan _and CT_FU _at the end of treatment was significantly correlated with the CC between CT_plan _and CT_FU _(p = 0.05): increased tumor shrinkage resulted in lower CC values. This correlation was not significant for differences between CT_plan _and CT_FU _acquired midway of the treatment (p = 0.15).

### Morphological pattern of tumor regression

**Figure 4:**
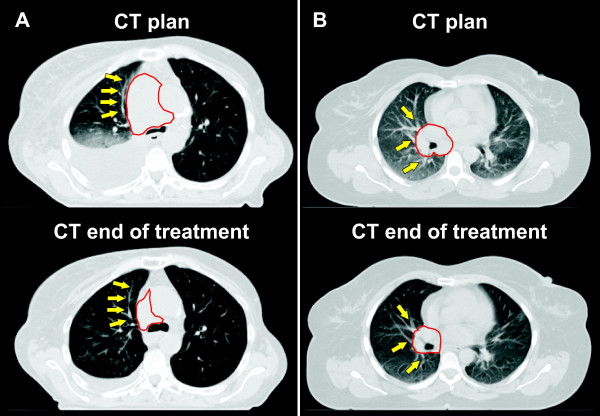
**Morphological patter of tumor shrinkage fora) patient # 11:** the tumor surrounding pulmonary tissue expanded in correlation with shrinkage of the tumor,** b) patient # 5:** tumor shrinkage released the pulmonary structures (bronchi and vessels). The contour of the macroscopic primary tumor is shown in red and arrows point to pulmonary landmark structures surrounding the tumor.

Visual evaluation of CT_plan _and CT_FU _acquired at the end of the treatment course regarding normal tissue landmark structures in the lung located close to the tumor showed inconsistent results. No suitable landmark structures were found in two patients. A morphological pattern of tumor shrinkage, where the pulmonary tissue expanded due to tumor shrinkage during the treatment course was observed in two patients; both tumors were located centrally (Fig. 4a). A pattern of tumor shrinkage, where the pulmonary tumor released vessels and bronchi during the treatment course was observed in four patients (Fig. 4b). A mixed regression pattern was observed in 5/13 patients.

### Visual evaluation of deformable image registration

CT_plan _and CT_FU _were not acquired with respiration correlated 4D-CT imaging and consequently were not captured in corresponding phases of breathing. This was corrected successfully by DR indicated by a close match of the diaphragm, chest wall and mediastinum. Also weight loss was corrected by DR indicated by a close match of the patients' outline; note that the patient's outline was used for calculation of the deformation map. Severe deformation artefacts were observed in three patients: a large pleural effusion resolved completely in two patients and a large atelectasis resolved in another patient. The shape of the target in the deformed image was affected in the patient with the resolved atelectasis.

**Figure 5:**
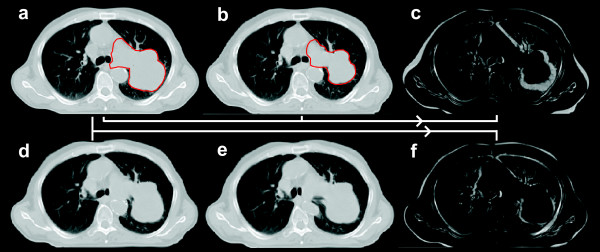
**Patient # 12: a) planning CT; b) follow-up CT at the end of treatment; c) difference image between planning CT and follow-up CT; d) deformed follow-up CT using all target and organs-at risk meshes; e) deformed follow-up CT using target, lung and spinal cord; f) difference image between planning CT and deformed follow-up CT based on all target organs-at risk meshes**. The contours of the macroscopic primary tumor in the planning CT and the follow-up CT are shown in red. Note the distortion of the vertebral body and the aorta without using meshes of these organs for deformable registration in e).

**Figure 6:**
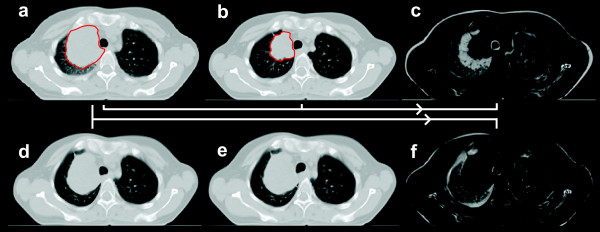
**Patient # 6: a) planning CT; b) follow-up CT at the end of treatment; c) difference image between planning CT and follow-up CT; d) deformed follow-up CT using all target and organs-at risk meshes; e) deformed follow-up CT using target, lung and spinal cord; f) difference image between planning CT and deformed follow-up CT based on all target organs-at risk meshes**. The contours of the macroscopic primary tumor in the planning CT and the follow-up CT are shown in red. Note the distortion of the vertebral body and the trachea without using meshes of these organs for deformable registration in e).

**Figure 7:**
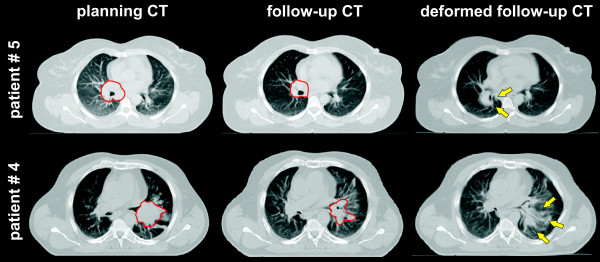
**Suboptimal results of deformable image registration**. The contours of the macroscopic primary tumor in the planning CT and the follow-up CT are shown in red and arrows point to artefacts after deformable image registration. Patient #5: deformation artefacts of the hilar bronchi (which were included into the GTV). The rather large amount of normal tissue within the GTV is probably responsible for this poor performance of DR. Patient #4: deformation artefacts after tumor shrinkage with a decay of the GTV.

Regarding the shape of the GTV, best visual results of the DR were observed in patients with large, solid tumors, which were clearly separated from the surrounding normal tissue in both CT_plan _and CT_FU_. Two examples of accurate DR are shown in fig. 5 and 6. Three situations caused significant deformation artefacts. In one patient, a resolving atelectasis could not be covered by segmentation and DR (described above). In one patient, the tumor was located around large bronchi and segmentation of these bronchi as normal tissue was not possible, because the structures were too small (Fig. 7, patient # 5). In the last case, no clear shrinkage but more a decay of the tumor was observed during the treatment course (Fig. 7, patient # 4).

The pulmonary tissue in close vicinity around the tumor showed moderate to severe deformation artefacts in all patients: application of the deformation matrix to CT_FU _expanded the GTV to the initial size in CT_plan _with the consequence of "compression" of the surrounding pulmonary tissue.

### Quantitative evaluation of deformable image registration

Comparison of CT_plan _and CT_deform _was performed for evaluation of the DR. Based on ROI_extended_, DR improved the CC for images acquired mid-way of the treatment course from 0.76 ± 0.11 to 0.88 ± 0.03. For CT images acquired at the end of treatment, a similar improvement was observed: CC increased from 0.74 ± 0.10 to 0.86 ± 0.05. Improvements in these CC values were observed for all 13 patients. Detailed results are shown in fig. 2.

If ROI_limited _was used for evaluation of the DR, the improvement in the similarity values was smaller compared to ROI_extended_. For images acquired mid-way of the treatment, DR improved CC from 0.64 ± 0.15 to 0.70 ± 0.15. However, similarity decreased for 2/13 patients. Similar findings were made for images acquired at the end of treatment: DR improved CC from 0.53 ± 0.16 to 0.62 ± 0.14. Decreased similarity was observed for 3/13 patients. Detailed results are shown in fig. 3.

The ratio r = CC (CT_plan _vs. CT_deform_)/CC (CT_plan _vs. CT_FU_) for ROI_limited _was significantly correlated with the volume of the GTV in CT_plan _(p = 0.03): an increased improvement in similarity due to DR was observed for larger GTV volumes. Additionally, a significant correlation between changes of the CC due to DR and absolute volume reduction of the GTV was observed (p = 0.02): improvement in similarity due to DR was larger for increased tumor shrinkage.

Manual landmark registration for evaluation of the accuracy of the DR was performed. Distances (3D vector) between corresponding landmark points on the GTV surface were 11.8 mm ± 5.1 mm for CT_plan _versus CT_FU _and these distances were reduced to 2.9 mm ± 1.2 mm for CT_plan _versus CT_FU _after DR was performed. However, in two patients the performance of the DR was not sufficient for reliable analysis of the GTV shape in CT_deform _and these two patients were excluded from the analysis above. Regarding the carina and vessel bifurcations, DR reduced the distances between corresponding landmark structures by about 40% on average; residual errors after DR ranged between 4 mm and 6 mm on average; detailed results are shown in table 2.

**Table 2:** Results of manual landmark registration for evaluation of the accuracy of the deformable image registration

	CT_plan _versus CT_FU_			CT_plan _versus CT_deform_		
	Average (mm)	StDev (mm)	90^th ^percentile (mm)	Average (mm)	StDev (mm)	**90^th ^percentile **(mm)
GTV	11.8	± 5.1	21	2.9	± 1.2	5.7

Carina	6.0	± 3.2	10	4.5	± 2.5	6.5

Pulmonary landmarks - same lobe as GTV	8.3	± 3.8	15	5.5	± 2.3	11

Pulmonary landmarks - different lobes as GTV	8.0	± 3.8	13	6.3	± 2.8	13

In general, good agreement between visual and quantitative analysis of DR was observed. However, poor CC values were observed in two patients despite good visual results regarding the shape of the GTV: an air-filled cavern developed within the GTV during radiochemotherapy in these two patients; DR successfully restore the GTV outline in these two patients but the inside of the GTV was soft-tissue in CT_plan _and partially air in CT_deform _resulting in poor CC.

## Discussion

The performance of different DR algorithms has been validated based on respiration correlated CT images in the thoracic region by a number of studies [15-24]. However, deformable image registration for advanced stage NSCLC with repeated CT images during the course of treatment is significantly more difficult for DR: regression of the tumor volume combined with weight loss of the patients and changes of atelectasis and pleural effusions make DR especially challenging. To our best knowledge, this is the first study evaluating the accuracy of DR in the context of such dramatic anatomical changes.

CT images acquired midway of the radiochemotherapy showed a decrease of the median GTV volume by almost 30% and the median GTV volume was reduced by more than 40% in CT images acquired at the end of treatment. This significant tumor regression is in good agreement with data in the literature [9,25-27]. In contrast, Bosmans et al. reported no decrease of the tumor volume in CT images acquired in the first and second week of radiotherapy on average for 23 patients, but a large heterogeneity was observed in this patient population [28]; similar findings were made for metastatic lymph nodes [29]. Clinically significant tumor regression was not observed by Siker et al, however, hypo-fractionated irradiation schemas were used in that study [30].

Overall, the surface-based algorithm of DR performed reasonable with large differences between patients. As expected, results of the DR were better for registration of the planning CT and CT images acquired mid-way of treatment compared to registration of planning CT and CT images acquired at the end of the treatment course. Differences between planning CT and follow-up CT images caused by patients' weight loss and different phases of breathing were managed well by the DR in all patients indicated by a close match of the mediastinum, chest wall, diaphragm and outline.

Manual landmark registration was performed for evaluation of the DR accuracy. Residual errors after DR were small at the GTV surface with 3D errors of 2.9 mm on average. Larger residual errors after DR were measured for intrapulmonary vessel bifurcations and the carina, where 3D errors ranged between 4.5 mm and 6.3 mm on average. These residual errors after DR are slightly larger compared to studies using surface-based DR in respiration correlated CT images [15,16]. However, results are realistic considering the tremendous anatomical changes observed in our study compared to the moderate anatomical changes usually observed in respiration correlated CT images. Studies using different DR algorithms for respiration correlated CT images reported residual errors of landmark registration ranging between 1 mm and 5 mm on average depending on the DR algorithm and type of landmark structures [17,18,20-22].

The surface-based DR algorithm has been validated on respiration correlated CT images of patients with pulmonary tumors and it has been described that segmentation of the GTV, lung, heart and spinal cord are sufficient for generation of the deformation map [31]. Our results suggest that more normal structures need to be segmented in adaptive radiotherapy during conventionally fractionated radiotherapy. The outline is certainly necessary to deal with weight loss of the patients. Additionally, segmentation of normal structures in close vicinity to the shrinking tumor was performed (vertebral bodies, trachea, aortic arch, sternum); otherwise the DR resulted in distortion of these normal structures. The effort for segmentation of these normal structures is acceptable because definition of organ models for automatic segmentation is straight forward.

For DR of the GTV itself, three situations are considered to be most problematic. First, for central tumors infiltrating the hilar and mediastinal structures, separate segmentation of tumor versus bronchi and vessels was not possible in all patients and these OARs were included into the GTV. This was done because the OARs were too small for manual and automatic segmentation or because separation of GTV and OARs was not possible in CT images without application of i.v. contrast or additional biological imaging. Consequences were deformation artefacts of these OARs after application of DR.

Second, a decay of the tumor with dissolving boundaries between tumor and lung is difficult for all methods of DR. Such a response pattern was observed in one of the patients. Reports in the literature mainly focused on volumetric analysis and did not describe such morphological changes of tumor regression. Adaptive approaches with shrinking irradiation fields are certainly not safe in these patients.

**Figure 8:**
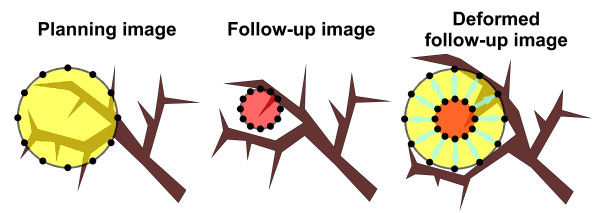
**Illustration of the consequences of an infiltrative growth pattern on deformable image registration: **left image) infiltrative growth pattern of the NSCLC (yellow with mesh points on the surface); middle image) tumor regression (red with mesh points on the surface) with release of the pulmonary structures; right image) transformation of the middle image into the left image based on the mesh points on the surface of the GTV: note the distortion of the pulmonary structures.

Third, a pattern of tumor regression with the pulmonary frame remaining stable was impossible to solve for the DR. This is illustrated in fig. 8. The tumor is growing in an infiltrative pattern within the pulmonary frame. During radiochemotherapy tumor regression occurs with the position of the pulmonary frame remaining stable. The algorithm of DR then expands the tumor to the original size and shape and the consequences are deformation artefacts with compression of the pulmonary tissue. Such a pattern of tumor shrinkage was observed at least partially in 9/13 patients. To cope with this issue, DR algorithms needed to know that one voxel can belong to two different organs with different corresponding deformation vectors.

Major disadvantage of this surface based DR method is the need for segmentation of all images. Auto-segmentation worked reasonable well for the OARs, however, manual delineation of the pulmonary tumor was required. All delineation work was performed by one single observer (MG) because large inter-observer variability in delineation of pulmonary tumors is a well known issue even if standardised protocols are defined [32,33]. Additionally, FDG-PET images were integrated into target definition, which has been shown to reduce delineation uncertainties significantly [34,35]. No method of GTV auto-contouring was applied because FDG-PET images were only available for treatment planning and because there still exist uncertainties about the best method for FDG-PET based auto-segmentation of lung tumors [36-38].

Some limitations of this study need to be addressed. 1) As discussed above, manual segmentation of the tumor was performed with the consequence of potential intra-observer variability. 2) CT images were acquired without application of 4D respiration correlated technique; this was done to limit radiation exposure of the patient by repeated CT imaging. However, this is not considered to influence results significantly. A 4D-CT was acquired at treatment planning for all patients and motion amplitude was ≤10 mm in 10 patients and maximum 14 mm. Consequently, motion artefacts due to free breathing during image acquisition are considered to be small using a 24-slice CT scanner. 3) Elastic body splines were used by the DR algorithm for interpolation within the organs. It has been shown that the application of algorithms based on appropriate tissue parameters improves the accuracy of this interpolation process [39]. However, exact parameters for all normal tissues are not available and it is unknown whether these parameters need to be patient-specific or not. 4) Macroscopic tumors are well known to be composed of subvolumes with different biological behaviour e.g. perfusion, hypoxia and proliferation. Consequently, homogeneous tumor shrinkage during radiochemotherapy is highly unlikely. However, this was not considered by the surface based DR in our study. Voxel-based DR algorithms would also not have been able to deal with this issue because biological characterization is not possible in CT images. Repeated biological imaging during radiotherapy and radiochemotherapy will be required for further analysis of this important aspect of adaptive radiotherapy.

We have recently established dose calculation in cone-beam CT images [40] and it is planned to use these cone-beam CT images in the process of adaptive radiotherapy. Consequently, DR between spiral CT images and cone-beam CT images needs to be analyzed as the next step.

## Conclusions

The surface-based DR performed accurately for the majority of the patients with locally advanced NSCLC and is considered as suitable for application in adaptive radiotherapy. Residual errors after DR were small at the surface of the GTV, but larger uncertainties within the lungs need to be considered.

## Competing interests

The authors declare that they have no competing interests.

## Authors' contributions

All authors read and approved the final manuscript.

MG designed the study, performed the analysis drafted and revised the manuscript.

KB developed in-house software for voxel-based analysis of CT image and participated in design of the study.

AR participated in the data analysis and revised the manuscript.

JW participated in the data analysis and revised the manuscript.

MF participated in the study design and revised the manuscript.
